# δ-Cells: The Neighborhood Watch in the Islet Community

**DOI:** 10.3390/biology10020074

**Published:** 2021-01-21

**Authors:** Rui Gao, Tao Yang, Quan Zhang

**Affiliations:** 1 Oxford Centre for Diabetes, Endocrinology and Metabolism, Radcliffe Department of Medicine, University of Oxford, Oxford OX3 7LE, UK; chinagaorui@hotmail.com; 2 Department of Endocrinology and Metabolism, The First Affiliated Hospital of Nanjing Medical University, Nanjing 210029, China; yangt@njmu.edu.cn

**Keywords:** pancreatic δ-cells, somatostatin, paracrine regulation, intra-islet coordination, diabetes

## Abstract

**Simple Summary:**

Pancreatic islets are micro-organs composed of several endocrine cell types, including α-cells (secreting glucose-elevating glucagon), β-cells (releasing glucose-lowering insulin) and δ-cells (producing somatostatin, a potent inhibitor of insulin and glucagon secretion). Despite their low percentage within the islets (~5%), δ-cells play an important role in maintaining a balanced hormone output. This is facilitated by their complex morphology projections enabling interaction with other endocrine cells. δ-cells are electrically excitable and, like in β-cells, K_ATP_ channels mediate the metabolic signals by modulating δ-cell membrane potential. However, Ca^2+^ signals amplified by mobilization of intracellular Ca^2+^ stores play a fundamental part in the process of glucose-induced somatostatin secretion and this can be independent of δ-cell electrical activity. Apart from their intrinsic regulatory mechanisms, δ-cells’ somatostatin secretion is tightly modulated by neighboring “non-δ-cells”, fulfilling its reciprocal feedback paracrine function. In this review, we summarize the structural features of δ-cells; the intracellular signaling of δ-cells in response to nutritional stimuli; and the molecular signals mediating the paracrine crosstalk between δ- and non-δ-cells. Finally, the function of δ-cells and their intercellular interactions are impaired in diabetes. Thus, restoring δ-cell function/signaling in diabetes would be a promising approach for developing novel treatment for diabetes.

**Abstract:**

Somatostatin-secreting δ-cells have aroused great attention due to their powerful roles in coordination of islet insulin and glucagon secretion and maintenance of glucose homeostasis. δ-cells exhibit neuron-like morphology with projections which enable pan-islet somatostatin paracrine regulation despite their scarcity in the islets. The expression of a range of hormone and neurotransmitter receptors allows δ-cells to integrate paracrine, endocrine, neural and nutritional inputs, and provide rapid and precise feedback modulations on glucagon and insulin secretion from α- and β-cells, respectively. Interestingly, the paracrine tone of δ-cells can be effectively modified in response to factors released by neighboring cells in this interactive communication, such as insulin, urocortin 3 and γ-aminobutyric acid from β-cells, glucagon, glutamate and glucagon-like peptide-1 from α-cells. In the setting of diabetes, defects in δ-cell function lead to suboptimal insulin and glucagon outputs and lift the glycemic set-point. The interaction of δ-cells and non-δ-cells also becomes defective in diabetes, with reduces paracrine feedback to β-cells to exacerbate hyperglycemia or enhanced inhibition of α-cells, disabling counter-regulation, to cause hypoglycemia. Thus, it is possible to restore/optimize islet function in diabetes targeting somatostatin signaling, which could open novel avenues for the development of effective diabetic treatments.

## 1. Introduction

The islets of Langerhans are a “heterogeneous community” formed by different types of endocrine cells and nonendocrine supporting cells. In addition to glucagon-secreting α-cells and insulin-producing β-cells, somatostatin-releasing δ-cells comprise only ~5% of the islet endocrine cells [1]. δ-cells have aroused great attention in the last decade due to their powerful roles coordinating islet hormone output and the maintenance of glucose homeostasis [2].

δ-cells have been considered the intra-islet local paracrine regulator of α- and β-cells. The peptide they secrete, somatostatin, is a potent and fast inhibitor for both insulin and glucagon secretion [3,4], the two glucose-regulating islet hormones. Appropriate somatostatin secretion can therefore effectively prevent possible oversecretion of insulin and glucagon, preventing large fluctuation of plasma glucose levels. Although small in number, δ-cells are able to communicate with the majority of α- and β-cells in the same islets, a remarkable ability that is attributable to their unique neuron-like morphology: the dendrite-like process can extend typically several cell lengths, forming a pan-islet paracrine network [5]. Ablation of this network can lead to severe hypoglycemia, impaired islet function and neonatal death in rodents [6], highlighting the functional importance of δ-cells in maintaining glucose homeostasis. Despite their role in islet function, how exactly δ-cells are regulated remains elusive. However, transcriptomic data from enriched δ-cell fractions revealed that δ-cells express a wide range of hormone and neurotransmitter receptors, including glucagon receptor (GCGR), glucagon-like peptide-1 receptor (GLP-1R), glutamate receptor 4 (GluR4) and growth hormone secretagogue receptor (GHSR) [7,8], which suggest the ability of δ-cells to sense paracrine, endocrine, neural and nutritional signals.

Given the potency of somatostatin in inhibiting glucagon and insulin secretion, it is not surprising that defects in δ-cell function and aberrant regulation of paracrine interactions play a fundamental role in the development of diabetes. For example, the loss of Urocortin3 (Ucn3) from β-cells at early stages of diabetes deprive δ-cells of the principal stimulatory factor for somatostatin release. The dysregulation of somatostatin-mediated feedback control between β- and δ-cells would increase glucose volatility [9]. Additionally, hypersecretion of somatostatin can disable the counter-regulatory glucagon secretion during insulin-induced hypoglycemia in diabetes [10]. Thus, somatostatin receptor (SSTR) antagonists could be applied as an adjunct to insulin therapy to lower the incidence of hypoglycemic events due to aggressive glycemic control [10,11].

In this review, we focus on the paracrine regulatory role of δ-cells from four different perspectives: (1) the structural basis of δ-cells; (2) the intracellular signaling of δ-cells in response to nutritional stimuli; (3) the bidirectional signals involved the paracrine crosstalks between δ- and β-cells, δ- and α-cells, and how they coordinate insulin and glucagon secretion; (4) how these interactions are affected in the pathophysiology of diabetes and potential pharmacological implication for glycemic management.

## 2. Structural Basis for δ-Cell Paracrine Regulation

**Figure 1:**
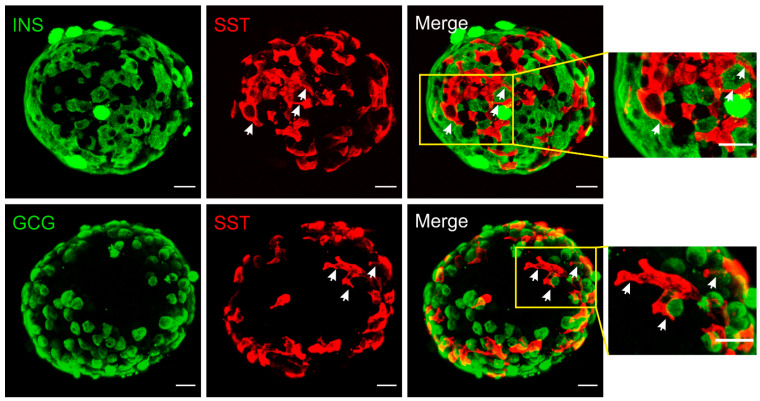
Immunofluorescent staining of mouse islets. Somatostatin (red), insulin (green, upper panels) or glucagon (green, lower panels). Merged panels are as indicated and areas in yellow rectangles are displayed on the right with an expanded scale. These reconstructed confocal images demonstrated the network of δ-cells and their proximity to α- and β-cells at the exterior of the islet. Note the morphology of δ-cells, which is distinctly different from α- and β-cells. The elongated projections are highlighted by arrows (white). Scale bar = 20 µm.

In rodent islets, δ-cells are situated in the outer islet mantle closer to α-cells, while in humans they are found scattered throughout the islets [12]. However, their proximity to blood vessels is well conserved cross-species. Different from the spherical α- and β-cells, mouse δ-cells exhibit neuron-like morphology with a well-defined cell soma and filopodia-like processes (Figure 1). The elongated projections (ranging from 2 to 27 μm [5,13]) are somatostatin-release competent and can compensate for the scarcity of δ-cells, enabling pan-islet somatostatin paracrine regulation. This morphological feature is not conserved in all species. In human islets, δ-cells have fewer axon-like projections and are significantly more compact than mouse δ-cells [14]. However, human δ-cells are scattered throughout the islets and are intermingled with α- and β-cells. Therefore, their pan-islet paracrine function is conserved in human islets. It is also intriguing that these projections (or filopodia) are highly plastic, showing rapid morphological change in response to vascular endothelial growth factor A (VEGF-A) and insulin-like growth factor 1 (IGF-1) [13]. Therefore, it is possible that the plasticity of δ-cells, together with their epigenetic adaptivity [15], contributes towards the changes of islet function in response to variable metabolic environment.

## 3. Nutritional Regulation of Somatostatin Secretion

### 3.1. Glucose

Due to the scarcity of δ-cells [1], the cellular regulatory mechanisms of somatostatin secretion remain largely unknown. The recent development of transgenic mice in which fluorescent proteins are specifically expressed in δ-cells (e.g., SST-tdRFP and SST-GCaMP3 mice [7,13,16,17,18]) enables in-depth studies of mechanisms that control somatostatin release. It has been established that glucose stimulates δ-cell somatostatin secretion via both membrane potential-dependent (“V_m_-dependent”) and -independent pathways (“V_m_-independent”) [17]. The V_m_-dependent pathway largely resembles that of the β-cells and involves glucose-induced reduction in K_ATP_ channel activity, membrane depolarization, action potential firing and Ca^2+^-dependent exocytosis [19]. Accordingly, somatostatin secretion can be stimulated by K_ATP_ channel blocker (e.g., tolbutamide) in the absence of glucose and inhibited by activation of K_ATP_ channel using diazoxide in the presence of glucose [20,21]. However, it is interesting that 70 mM K^+^ or tolbutamide alone evokes less somatostatin secretion than high glucose alone, despite a stronger depolarizing effect, indicating that glucose exerts additional stimulatory effects on somatostatin secretion at a level that is distal to electrical activity (i.e., the V_m_-independent pathway) [17,21]. Earlier work identified that the majority of glucose-induced somatostatin secretion (GISS) depends on Ca^2+^-induced Ca^2+^ release (CICR) from the endoplasmic reticulum (ER) through the ryanodine receptor 3 (RyR3) Ca^2+^-release channels [21]. More recently, we identified that activating sodium-glucose cotransporters can rapidly increase intracellular Na^+^ concentration, which in turn triggers CICR through activation of intracellular Na^+^-Ca^2+^ exchange without evoking electrical activity [22]. However, it is likely that the full scale CICR and somatostatin secretion may occur only following glucose metabolism. This is because that sensitization of RyR3 and exocytotic machinery in δ-cells requires elevation of intracellular cyclic AMP (cAMP) induced by glucose metabolism [17].

**Figure 2:**
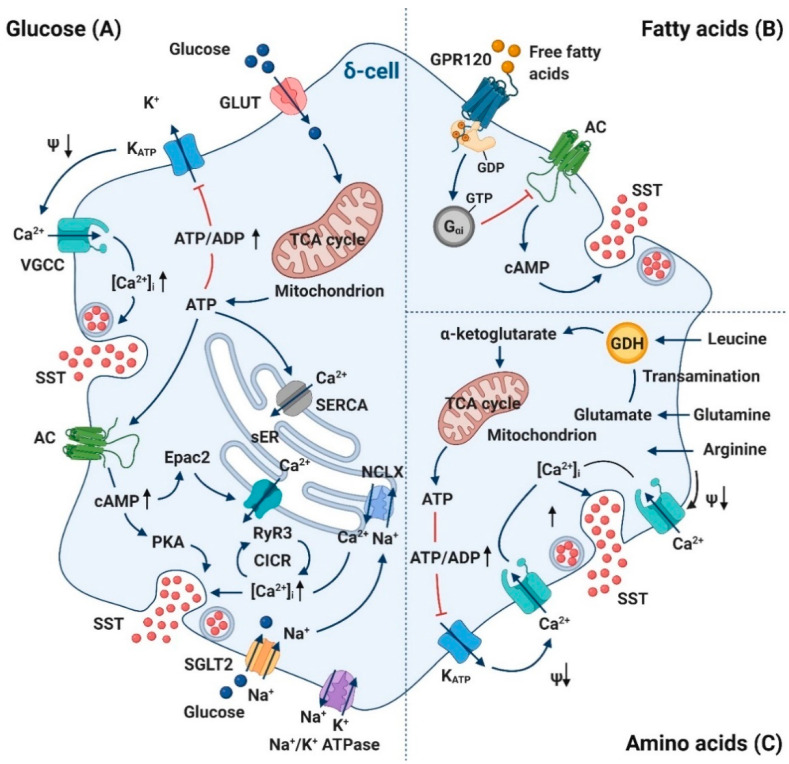
Nutrient-secretion coupling in δ-cells. (**A**) Glucose stimulates δ-cell somatostatin secretion via both membrane potential-dependent (“V_m_-dependent”) and -independent pathways (“V_m_-independent”). V_m_-dependent pathway: Glucose metabolism increases cytoplasmic ATP/ADP ratio and consequently closes K_ATP_ channels. The subsequent membrane depolarization leads to opening of voltage-gated Ca^2+^ channels (VGCC) and influx of [Ca^2+^]_I_ to trigger exocytosis. V_m_-independent pathway: ATP production stimulated by glucose metabolism restores luminal ER calcium levels through SERCA pump and elevates intracellular cAMP via adenylyl cyclase (AC). Whereas cAMP as a second messenger of PKA directly potentiates δ-cell exocytosis, its effect on activation of ryanodine receptor 3 (RyR3) and sensitization of calcium-induced calcium release (CICR) in sER is mediated by Epac2. Glucose also promotes somatostatin secretion by increasing [Na^+^]_i_ via sodium-dependent glucose cotransporter 2 (SGLT2) and thereby increases [Ca^2+^]_i_ through stimulation of intracellular Na^+^-Ca^2+^exchange (NCLX). The resultant elevation in [Ca^2+^]_i_ triggers somatostatin secretion. (**B**) Free fatty acids inhibit somatostatin release by binding to non-esterified fatty acids (NEFA)-responsive G-protein coupled receptor 120 (GPR120) on δ-cells. Its downstream G_αi_ inhibits AC activity and decreases cAMP production, which in turn results in reduced activity of PKA. (**C**) The regulatory role of amino acids in δ-cells might resemble their actions in β-cells. We postulate that arginine could stimulate somatostatin secretion via electrogenic transport, thereby increasing membrane depolarization and [Ca^2+^]_i_ through opening of voltage-gated Ca^2+^ channels, while leucine-induced somatostatin secretion might involve transamination of leucine to α-ketoisocaproate, entry into tricarboxylic acid cycle (TCA) via acetyl-CoA, ATP generation and closure of K_ATP_ channels. GLUT, glucose transporter; sER, smooth endoplasmic reticulum; SERCA, sarco/endoplasmic reticulum Ca^2+^-ATPase; cAMP, cyclic AMP; PKA, protein Kinase A; VGCC, voltage-gated Ca^2+^ channel; GDH, glutamate dehydrogenase; SST, somatostatin. Created by BioRender.com.

Whereas both V_m_-dependent and -independent pathways synergistically contribute to a full stimulatory effect of somatostatin secretion by glucose (Figure 2A), it is interesting to note that δ-cell electrical activity plays a relatively minor role somatostatin release. This is physiologically relevant given the potency of somatostatin in inhibiting insulin and glucagon secretion [3,4]. δ-cells have very high input resistance (comparable to that of β-cells at high glucose; own unpublished observation) even in the absence of glucose or cAMP and therefore accidental action potential firing could easily occur. The requirement of the V_m_-dependent pathway for concentrated somatostatin release thus could provide “safety” to avoid unwanted “spillage” of somatostatin.

### 3.2. Fatty Acids

The free fatty acid palmitate is a strong inhibitor of glucose-induced somatostatin release. Palmitate at 0.5–1 mM can efficiently decrease GISS by 50% [23]. This effect is likely to be mediated by the non-esterified fatty acids (NEFA)-responsive G-protein coupled receptor 120 (GPR120), which is preferentially expressed in δ-cells of both mouse and human islets. This is confirmed by pharmacological study using a range of selective GPR120 agonists and GPR120-knockout mice [24]. The effect of GPR120 could be mediated by G_αi_ through adenylate cyclase (AC) inhibition to reduce cAMP production. This inhibitory effect of palmitate on δ-cells could relieve α- and β-cells from paracrine suppression, contributing to the palmitate-induced augmentation in insulin and glucagon secretion [23,25] (Figure 2B).

### 3.3. Amino Acids

Amino acids, especially arginine and leucine, can enhance δ-cell somatostatin secretion in isolated perfused pancreas [26,27,28]. The exact mechanisms by which amino acids stimulate somatostatin remain to be established, but δ-cells express at least several amino acid transporters at high level. These include cationic amino acid transporters *Slc7a1* (encodes CAT-1), *Slc7a2* (encodes CAT-2) and neutral amino acid transporter *Slc7a5* (encodes LAT1) [8]. It is postulated that amino acids stimulate somatostatin via mechanisms that are similar to that reported in β-cells [29]. Electrogenic transport (e.g., arginine) could induce δ-cell membrane depolarization and electrical activity, while leucine-induced somatostatin secretion might involve transamination of leucine to α-ketoisocaproate, entry into tricarboxylic acid cycle (TCA) cycle via acetyl-CoA, ATP generation and closure of K_ATP_ channels (Figure 2C).

## 4. β-Cell-Mediated Paracrine Regulation of δ-Cells

### 4.1. Insulin

Whereas the paracrine inhibitory effect of somatostatin on insulin secretion has been well established, whether insulin is a reciprocal regulator of δ-cell function remains controversial over the last few decades. In isolated islets, administration of exogenous insulin has been reported to have no effect in mouse [30] or rat [31]. The lack of detectable responses to exogenous insulin could be attributed to saturated endogenous interstitial insulin concentrations within islets. In a perfused pancreas, however, the result is even more inconclusive with a stimulatory effect in chicken [32], an inhibitory effect in rat [26] or no effect in canine [33]. Immunoneutralization of endogenous insulin with specific antibodies was also shown to increase somatostatin release in an anterograde-infused mouse pancreas [34] but reduce somatostatin secretion in a perfused human pancreas [35]. These contradictions may be attributed to species-dependent differences in islet architecture [12] or different experimental models employed (perfusion rate and direction).

**Figure 3:**
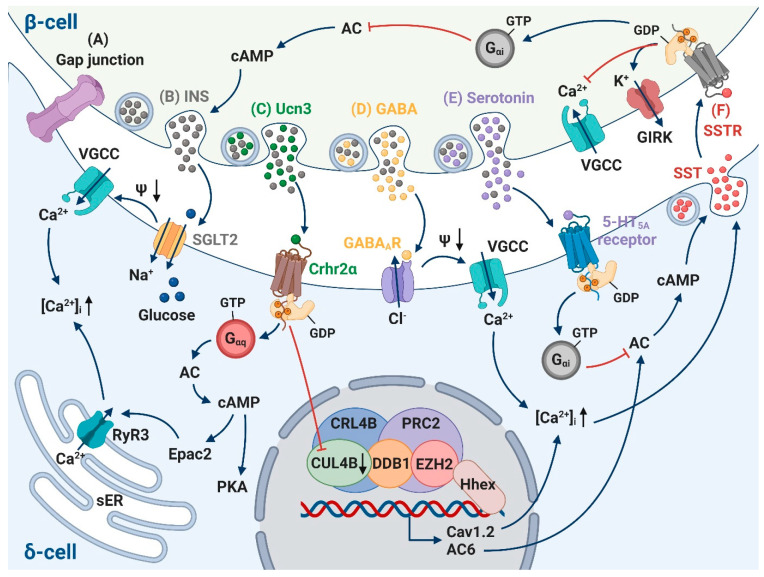
Paracrine interaction between β-cells and δ-cells. (**A**) δ-cells are directly coupled to β-cells via gap junction. (**B**) Insulin (grey) secreted by β-cells promotes somatostatin secretion via stimulation of sodium-dependent glucose cotransporter 2 (SGLT2). Na^+^-dependent glucose uptake induced by SGLT2 triggers a small depolarizing current and leads to action potential firing and opening of VGCC. (**C**) Urocortin3 (Ucn3, green, co-secreted with insulin, which is marked as grey) potentiates somatostatin release by activating type 2 corticotropin releasing hormone receptor (Crhr2) on δ-cells. The action of Ucn3 is mediated through both the cAMP-dependent pathway and epigenetic regulation of a super-complex comprising cullin 4B-RING E3 ligase (CRL4B) and polycomb repressive complex 2 (PRC2). In response to Ucn3 stimulation, the decreased expression of cullin 4B (CUL4B) and subunit histone-lysine N-methyltransferase EZH2 releases the repressive role of the CRL4B-PRC2 complex in the expression of Cav1.2 and adenylyl cyclase 6 (AC6), thereby increasing levels for second messengers Ca^2+^ and cAMP and ultimately leading to increased somatostatin secretion. (**D**) γ-Aminobutyric acid (GABA, yellow, co-secreted with insulin, which is marked as grey), via GABA_A_ receptors (GABA_A_Rs), induces membrane depolarization and facilitates Ca^2+^ entry via VGCCs. (**E**) Upon serotonin (purple, co-secreted with insulin, which is marked as grey) binding, 5-hydroxytryptamine receptor 5A (5-HT_5A_ receptor) signals via G_αi_ to inhibit AC, thus diminishing intracellular cAMP and inhibiting somatostatin secretion. (**F**) The downstream signaling of somatostatin (SST, red) activation of somatostatin receptor (SSTR) on β-cells involves activating the coupled G_αi_ protein leading to decreased cytoplasmic levels of cAMP; hyperpolarizing the membrane potential and inhibiting action potential firing via G protein-coupled inwardly rectifying K^+^ (GIRK) channels; inhibiting Ca^2+^ influx through voltage-gated P/Q-type Ca^2+^ channels. RyR3, ryanodine receptor 3; cAMP, cyclic AMP; PKA, protein Kinase A; sER, smooth endoplasmic reticulum; INS, insulin; SST, somatostatin. Created by BioRender.com.

A recent study revealed that ablating the insulin receptor in δ-cells results in lower somatostatin release and α-cell insensitivity to insulin, indicating that the inhibition of α-cells by insulin is mediated indirectly by stimulation of intra-islet somatostatin release [18]. The operation of sodium-glucose cotranporter-2 (SGLT2) was further identified to participate in this stimulatory effect of insulin on somatostatin secretion. Na^+^-dependent glucose uptake through SGLT2 could trigger a small depolarizing current and lead to action potential firing at least in some cells with sufficiently high SGLT2 expression and/or low K_ATP_ channel activity [18] (Figure 3B).

### 4.2. Urocortin3

Normal GISS requires Ucn3, a peptide hormone that is highly expressed in β-cells [9]. Ucn3 is co-released with insulin [36] and could potentiate somatostatin release via type 2 corticotropin releasing hormone receptor (Crhr2) on δ-cells [9]. Acute blockade of endogenous Ucn3 using Crhr2-selective antagonist Astressin2B in wild-type islets leads to an instant (minutes) reduction of somatostatin secretion and concomitant increase in insulin release [9]. These findings were also validated by genetically modified Ucn3-null islets and Crhr2-null islets that display relative δ-cell deficiency, selective reduction in somatostatin content and impaired basal and GISS compared to wild-type islets. The impaired somatostatin release further enables exaggerated first- and second-phase glucose-stimulated insulin secretion, which can be immediately normalized upon perfusion of synthetic Ucn3 peptide [9].

Continuous Ucn3 stimulation could also trigger long-term (hours) epigenetic modifications. The precise mechanism underlying this effect was found to be mediated via a super-complex comprising cullin 4B-RING E3 ligase (CRL4B) and polycomb repressive complex 2 (PRC2). In response to sustained Ucn3 stimulation, the decreased expression of cullin 4B (CUL4B) and subunit histone-lysine N-methyltransferase EZH2 releases the repressive role of CRL4B-PRC2 complex in the expression of Cav1.2 and adenylyl cyclase 6 (AC6), thereby increasing levels for second messengers Ca^2+^ and cAMP in pancreatic δ-cells and ultimately leading to increased somatostatin secretion [15,37]. Taken together, these observations concluded that Ucn3 activates a β-cell-dependent, δ-cell-mediated negative feedback loop which is essential for timely attenuation of excessive insulin release and avoiding volatile plasma glucose levels to ensure metabolic homeostasis (Figure 3C).

### 4.3. Gap Junction

Gap junction coupling of β-cells by connexin-36 (Cx36) is fundamental for the coordination of [Ca^2+^]_i_ oscillations, both by generating pulsatile insulin secretion in isolated islets [38,39] and affecting insulin secretion dynamics in vivo [40]. δ-cells also express Cx36 [8] and could in principle form a gap junction with adjacent β-cells. It has been proposed that β-and δ-cells’ electrical coupling underlies the synchronicity of insulin and somatostatin secretion pulses under the stimulation of glucose [41]. This theory was further developed by the implementation of an optogenetics technique. Light radiation of islets that express channelrhodopsin-2 specifically in β-cells (RIP-ChR2) could trigger β-cell membrane depolarization, an electrical signal that propagates to the δ-cells and, in turn, results in suppressed α-cells’ activity [16]. However, δ-cells can be activated at glucose concentrations that are subthreshold for β-cell activation (3 mM vs. 7 mM in mouse islets [42]) possibly due to different characteristics of K_ATP_ channels [19], and there is a 30 s delay between the pulses of somatostatin and insulin release [43]. These indicate the interaction between δ- and β-cells is not solely mediated by electrical coupling—chemical coupling of the two cell types could play a more profound role in this crosstalk (Figure 3A).

### 4.4. γ-Aminobutyric Acid

γ-Aminobutyric acid (GABA) is a classic inhibitory neurotransmitter in the mammalian central nervous system (CNS) and is synthesized from glutamic acid by glutamic acid decarboxylase (GAD). It is highly enriched in β-cells and is localized in both “synaptic”-like microvesicles [44,45] and insulin-containing large dense core vesicles [46]. GABA released from β-cells inhibits glucagon secretion in rodent islets by activating GABA_A_ receptors (GABA_A_Rs) to induce repolarization in α-cells [47,48]. Comparing that to the α-cells, there is only a handful of studies conducted regarding its effect on δ-cells to date. Patch-clamp experiments demonstrated that GABA_A_R is highly expressed on δ-cells (GABA-triggered Cl^-^ current is ~10-fold larger than that of the β-cells) and application of exogeneous GABA strongly depolarized δ-cells because of their high intracellular Cl^−^ concentration [46]. Therefore, GABA released from β-cells could contribute to the stimulation of somatostatin secretion at high glucose, and blockade of GABA_A_R could decrease somatostatin secretion [46]. This seemingly paradoxical effect of GABA on α-cells and δ-cells is probably due to the difference in intracellular Cl^-^ concentration (which determines the Cl^-^ reversal potential) and, of course, the possibility that GABA-induced α-cell repolarization may actually be a consequence of δ-cell activation (Figure 3D).

### 4.5. Serotonin

Serotonin is co-released with insulin from β-cells and has been investigated extensively as an autocrine signal regulating β-cell function and proliferation [49,50,51,52], while its effects on paracrine regulation of other islet endocrine cells are less investigated. Studies during the last decade concluded that serotonin as one of the paracrine signals inhibits neighboring α-cells and δ-cells [53]. After binding to G_αi_-coupled 5-hydroxytryptamine receptor 5A (5-HT_5A_ receptor) which is primarily expressed on δ-cells, serotonin could lower cAMP concentration and decrease somatostatin secretion in a concentration-dependent manner [53] (Figure 3E).

## 5. α-Cell-Mediated Paracrine Regulation of δ-Cells

### 5.1. Glucagon

The favorability of δ- and α-cell crosstalk was first suggested by Orci et al. based on their proximity in the islets across species [54]. While blockade of glucagon signaling with glucagon antibodies was found to reduce somatostatin release [31,35], application of exogeneous glucagon could stimulate somatostatin secretion both in isolated islets [31,55] and perfused mouse pancreas [56]. Diphtheria toxin-induced ablation of glucagon-producing cells can lead to decreased somatostatin secretion in response to different stimuli [56]. Glucagon receptor (GCGR) and glucagon-like peptide 1 receptor (GLP-1R) are expressed in δ-cells [8] and both could be activated by glucagon to mediate this paracrine regulation. Indeed, glucagon-stimulated somatostatin secretion could be abolished by GCGR or GLP-1R antagonists [56]. Thus, it is possible that a reciprocal feedback cycle operates between δ- and α-cells—glucagon stimulates somatostatin release which, in turn, inhibits glucagon secretion. It remains to be established how this interaction functions at even lower concentrations of glucose (e.g., 1 mM), where maximum glucagon secretion is observed.

**Figure 4:**
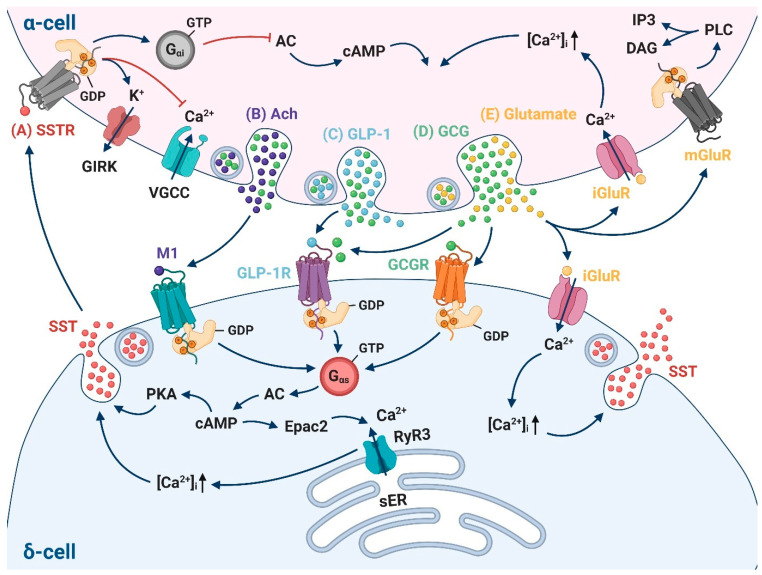
Paracrine interaction between α-cells and δ-cells. (**A**) The downstream signaling of somatostatin (SST, red) activation of somatostatin receptor (SSTR) on α-cells involves activating the coupled G_αi_ protein, leading to decreased cytoplasmic levels of cAMP; hyperpolarizing the membrane potential and inhibiting action potential firing via G protein-coupled inwardly rectifying K^+^ (GIRK) channels; inhibiting Ca^2+^ influx through voltage-gated P/Q-type Ca^2+^ channels. (**B**–**D**) The stimulatory effect of Ach (purple, co-secreted with glucagon, which is marked as green), GLP-1 (blue, co-secreted with glucagon, which is marked as green) and GCG (green) on somatostatin secretion is mediated via Ach receptor M1, GLP-1R and GCGR, respectively. All these receptors are G_αs_-coupled receptors which subsequently activate adenylate cyclase (AC), increase cAMP production and trigger PKA-potentiated δ-cell exocytosis. (**E**) Glutamate (yellow) is co-secreted with glucagon (green). In α-cells, it acts as an autocrine feedback signal potentiating adequate glucagon release through ionotropic glutamate receptors (iGluRs), whereas its inhibitory role is conducted via metabotropic glutamate receptors (mGluRs). In δ-cells, the pathways following the activation of iGluR might share some similarities with glutamate effect on α-cells, including membrane depolarization, opening of voltage-gated Ca^2+^ channels (VGCC), increase in cytoplasmic [Ca^2+^]_i_ and enhanced somatostatin secretion. RyR3, ryanodine receptor 3; cAMP, cyclic AMP; PKA, protein Kinase A; PLC, phospholipase C; IP3, inositol trisphosphate; DAG, diacyl glycerol; sER, smooth endoplasmic reticulum; Ach, acetylcholine; GLP-1, glucagon-like peptide-1; GLP-1R, glucagon-like peptide-1 receptor; GCG, glucagon; GCGR, glucagon receptor; SST, somatostatin. Created by BioRender.com.

Downstream signaling of the glucagon effect has not been investigated in-depth. Both GCGR and GLP-1R belong to the same family of G protein-coupled receptors (GPCRs) [42]. Glucagon binding to these G_αs_-coupled GPCRs can activate AC to elevate intracellular cAMP concentrations and subsequently trigger protein kinase A (PKA) and/or Epac2-potentiated δ-cell secretion. Additionally, it is unlikely that the effect of glucagon on somatostatin secretion is indirectly mediated through glucagon-induced insulin secretion [57]. The administration of the insulin receptor antagonist S961 affects neither basal nor glucagon-stimulated somatostatin release [56] (Figure 4D).

### 5.2. Glutamate

Glutamate is a major excitatory neurotransmitter in the CNS and it also plays an important role in modulating the function and viability of islet cells [58]. Glutamate is co-secreted with glucagon from α-cells under low glucose conditions and acts as a primary intercellular messenger [59]. In the islets, glutamate can be excitatory (through activation of ionotropic glutamate receptors (iGluRs) [60]), or inhibitory (via metabotropic glutamate receptors (mGluRs) [61]). For example, glutamate binding to iGluRs of the α-amino-3-hydroxy-5-methyl-4-isoxazolepropionic acid (AMPA)/kainate type on α-cells can act as an autocrine feedback loop to potentiate its own secretory activity and guarantee adequate glucagon release [62].

δ-cells express the AMPA-type receptor (GluR4), and glutamate was reported to trigger somatostatin secretion under low-glucose conditions [63], an effect that could be reversed by 6-cyano-7-nitroquinoxaline-2,3-dione (CNQX), an antagonist of the AMPA-type glutamate receptor. How glutamate stimulates δ-cells remains to be established at the single-cell level, but given the ionotropic property of GluR4, it is likely that glutamate induces δ-cell membrane depolarization, electrical activity and opening of voltage-gated Ca^2+^ channels to trigger δ-cell exocytosis and somatostatin secretion (Figure 4E).

### 5.3. Glucagon-Like Peptide-1

The effect of GLP-1 on β-cells relies on the activation of highly expressed GLP-1R and thereby, potentiation of glucose-induced insulin secretion when glucose reaches a certain threshold [64,65]. However, the function of GLP-1 in δ- and α-cell communication remains uncertain/controversial. The expression of GLP-1R is relatively high in δ-cells compared to α-cells [66], with only approximately 1% α-cell population presenting GLP-1R [67]. It therefore has been proposed that the inhibitory action of GLP-1 on α-cells is indirectly mediated via paracrine interaction of somatostatin released from neighboring δ-cells. This is based on the observation that a decrease in glucagon secretion by GLP-1 is invariably accompanied by stimulation in somatostatin release, and specific blockade of SSTR2 completely eliminates the glucagon-reducing effect of GLP-1 [30,68,69]. This phenomenon was also described in GLP-1R agonist liraglutide [70], which may account for about half of its glucose-lowering activity [71]. GLP-1R, as a G_αs_-coupled GPCR, could potentiate somatostatin secretion through AC activation and the following cAMP-mediated signaling cascade (Figure 4C).

### 5.4. Acetylcholine

In mouse islets, acetylcholine originates from intra-islet parasympathetic innervation [72]. By contrast, in human islets, α-cells provide paracrine cholinergic input to surrounding endocrine cells, sensitizing β-cells to respond optimally to subsequent increases in glucose concentration [73]. Endogenous acetylcholine does not only stimulate β-cell insulin secretion via muscarinic acetylcholine receptors M3 and M5, but also promotes δ-cell somatostatin release via receptor M1 [72,74,75]. This action can be abolished by atropine and stimulated by blocking acetylcholine degradation with physostigmine, suggesting that somatostatin secretion could be activated by endogenous cholinergic signaling. Indeed, further depleting releasable α-cell acetylcholine using an inhibitor of vesicular acetylcholine transporter eliminates the reversing effect of atropine, indicating acetylcholine released from α-cells stimulates somatostatin secretion [75]. Consequently, intra-islet cholinergic input exerts both positive and negative regulation on insulin secretion, stimulating directly by M3 receptor in β-cells and inhibiting indirectly by M1 receptor in δ-cells with increased somatostatin release (Figure 4B).

### 5.5. Glicentin-Related Pancreatic Polypeptide

In α-cells, proglucagon processing yields glucagon along with glicentin-related pancreatic polypeptide (GRPP) and a nonbiologically active proglucagon fragment consisting of amino acids 72–158 [76]. GRPP, as a product from proglucagon precursor, is packed in the same secretory granules as glucagon and co-secreted from α-cells in response to physiological stimuli. It is possible that GRPP may serve as a paracrine signaling peptide to regulate neighboring δ-cell function. Previous studies observed that porcine GRPP inhibits glucagon secretion in perfused canine pancreas [77,78] and rat GRPP elicits potent inhibition of glucose-stimulated insulin secretion from rat pancreas [79]. These effects were suggested to be, in part, a result of GRPP-stimulated somatostatin secretion. However, whether somatostatin receptor antagonists could reverse GRPP’s inhibitory action is yet to be demonstrated.

## 6. δ-Cell-Mediated Intra-Islet Coordination

### 6.1. Somatostatin

There are two types of somatostatin: a 14 amino acid form (SST-14) and a 28 amino acid form (SST-28), both proteolytically cleaved from a larger precursor. SST-14 is secreted primarily by somatostatin-expressing neurons and pancreatic δ-cells, while SST-28 is the dominant isoform released from D-cells of the gastrointestinal tract [2]. The fact that the circulating somatostatin concentration (~5–25 pM) is unaffected by pancreatectomy [80] and below the IC_50_/EC_50_ of intra-islet SSTRs [81,82] suggests that the gastrointestinal tract is responsible for the circulating somatostatin. Together with the fact that somatostatin degrades rapidly, it is assumed that δ-cells are the major source of somatostatin within the islets. δ-cells are active throughout a wide physiological range of glucose concentration (3–20 mM) [2]. This characteristic enables δ-cells to exert their regulatory function on both α- and β-cells to coordinate pancreatic islet hormone output precisely.

In mouse β-cells, it was previously believed that the most abundant form of SSTR is SSTR5 [83,84]. Recent RNA sequencing data using fluorescence-activated cell sorting (FACS)-purified mouse islet cell populations revealed that SSTR3 predominantly confers somatostatin-dependent inhibition of insulin secretion [7,8]. However, in human β-cells, a combination of SSTR1, SSTR2, SSTR3 and SSTR5 has been detected [85]. Somatostatin effect on α-cells is primarily mediated by SSTR2 both in mouse and human islets as unequivocally reported by different studies [7,8,86,87]. These GPCRs could inhibit their targeted cells via several mechanisms [86,88]: (1) activation of the G_αi_-coupled protein leading to decreased cytoplasmic levels of cAMP; (2) hyperpolarizing the membrane potential and inhibiting action potential firing via G protein-coupled inwardly rectifying K^+^ (GIRK) channels; (3) inhibiting Ca^2+^ influx through voltage-gated Ca^2+^ channels (Figure 3F and Figure 4A). The consistency that both somatostatin and insulin releases are stimulated linearly in a dose-dependent fashion by glucose is instrumental in preventing insulin-induced hypoglycemia [14]. Nevertheless, how δ-cells manage to control excessive glucagon secretion, considering only a small quantity of somatostatin is released at low glucose, remains unsolved. It is possible that the tight connection between α- and neighboring δ-cells facilitates a precise fine-tune mechanism of somatostatin locally and sporadically modulating α-cell actions.

Interestingly, both SSTR1 and SSTR3 are also found highly expressed in mouse δ-cells [7,8]. Together with the observation that SSTR antagonists could promote somatostatin secretion [69], δ-cells might have autocrine feedback inhibition restraining uncontrolled somatostatin release.

### 6.2. Neuronostatin

Neuronostatin is a recently identified peptide produced in the same tissues as somatostatin, including pancreatic δ-cells [89]. Although neuronostatin and somatostatin are derived from the same preprohormone, the effect of neuronostatin is not mediated by any of the five known SSTRs [90]. It has been described that neuronostatin could stimulate glucagon production and indirectly attenuate insulin secretion in low-glucose conditions through GPR107, which is abundantly expressed in α-cells [91]. Neuronostatin binding to GPR107 increases cAMP-independent PKA phosphorylation and proglucagon mRNA accumulation [92]. Further elucidations are required to demonstrate the physiological role of neuronostatin in response to different stimuli and whether its alternation participates in the development of diabetes.

### 6.3. Cortistatin

Despite being encoded by a different gene (*Cort*), cortistatin is a neuropeptide that shares structural similarities with somatostatin [93]. Other than its ability to activate SSTRs, it has also been determined that cortistatin can bind to GHSR [93,94]. Exposure of β-cells to cortistatin would lead to membrane potential hyperpolarization via activation of GIRK channels, reduction in glucose-induced action potentials and thus inhibition of insulin secretion [95]. This action can be reversed by SSTR antagonists. Cortistatin was found to be expressed in mouse and human islets, especially localized in δ-cells at both transcriptomic and protein levels [95,96,97,98]. However, in contrast, recent transcriptomic studies pointed to a minimal cortistatin expression level in purified mouse islets cells [7,8].

## 7. Paracrine Crosstalk of δ-Cells in Diabetes

Diabetes has long been recognized as a “bihormonal disorder”, characterized by glucagon excess in the setting of insulin deficiency or insulin resistance [99]. However, in recent years, δ-cells have been considered as a signaling hub integrating the inputs from nutrients, hormones and neurotransmitters, and then providing direct instructions on α- and β-cells by paracrine modulation of insulin and glucagon secretion [14]. Thus, it is possible that defects in the regulation of somatostatin secretion from δ-cells contribute to the pathogenesis of diabetes.

Single-cell transcriptomics has identified several differentially regulated genes in δ-cells between diabetics and nondiabetics, indicating the potential role of δ-cells in diabetic pathogenesis [100,101,102]. The expression patterns of SSTRs in islet cells are altered in type 2 diabetes mellitus (T2DM), with diminished levels of SSTR1 and SSTR4 in α-cells and SSTR1-4 in δ-cells [103], which could further contribute to impaired somatostatin paracrine regulation in diabetes. Morphological alternations and cytoarchitectural remodeling of islets can be observed in db/db diabetic mice with an initial increase in δ-cell numbers per islet around 8–10 weeks followed by a decrease around 20 weeks [104]. This suggests the islet paracrine tone changes throughout the course of diabetes development. The reduction in δ-cell population in diabetes was likewise observed in other species. In human and nonhuman primate diabetes, a marked reduction of β-cells, a relative/absolute increase in α-cells and a decrease in δ-cells have been identified [105,106]. The changes in δ-cell percentage correlate inversely with baseline glucagon levels [106], which might suggest that insufficient somatostatin production accounts for uncontrolled glucagon secretion. In patients with T2DM, δ-cells also experience several ultrastructural alternations as examined by electron microscopy, including degranulation, vacuolization and nuclei with condensed chromatin [105]. Additionally, prediabetic and diabetic animals exhibit a significant impairment of δ-cell activity, featured with reduced somatostatin release [107,108], lower [Ca^2+^]_i_ peak amplitudes and a higher percentage of unresponsive cells [13]. Interestingly, previous studies of db/db mice have shown apparent migration of δ-cells from a “capped” region of the islet periphery adjacent to vascular and/or ductal structures into the islet core, and a significant increase in β- and δ-cell contacts by augmented density of δ-cell projections [104]. Increased δ-cell filopodia length has also been described in high-fat-diet (HFD) prediabetic mice [13], compensating for the dysfunction of δ-cells to maintain contact with neighboring α- and β-cells.

**Figure 5:**
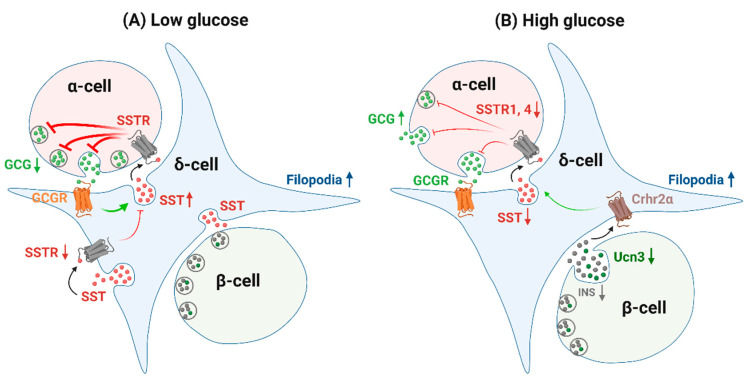
Paracrine crosstalk of δ-cells in diabetes. The expressions of somatostatin receptors (SSTRs) in islet cells are altered in diabetes with diminished levels of SSTR1 and SSTR4 in α-cells and SSTR1-4 in δ-cells. δ-cell filopodia length is increased in prediabetic and diabetic animal models to expand its contacts with α- and β-cells. (**A**) At low glucose, excessive somatostatin secretion is responsible for the defective counter-regulation of α-cells in diabetes with inadequate glucagon secreted in response to hypoglycemia. This may be due to the higher sensitivity of δ-cell to neighboring α-cell activity. (**B**) At high glucose, due to β-cell depletion and consequently reduced Ucn3 and insulin in diabetes, δ-cells lack the stimuli for sufficient glucose-induced somatostatin secretion (GISS), which could further lead to inappropriate glucagon release during high glucose. Green arrows indicate stimulation and red blunted arrows indicate inhibition. Line strength indicates strength of stimulation/inhibition. Note that the stimulation/inhibition of SST secretion is relative to its secretion at either low or high glucose under normal physiological conditions. GCGR, glucagon receptor; Crhr2, type 2 corticotropin releasing hormone receptor; SST, somatostatin; GCG, glucagon; INS, insulin; Ucn3, Urocortin3. Created by BioRender.com.

δ-cells are capable of precisely balancing insulin and glucagon output and determining the homeostatic set-point for plasma glucose. In prediabetes and diabetes, impaired δ-cell function can possibly lead to imbalance between α- and β-cells and shift the glycemic set-point towards hyperglycemia. It is also worth noticing that δ-cell paracrine crosstalk with α- and β-cells is also affected in diabetes. Regardless of autoimmune attacks in type 1 diabetes mellitus (T1DM) or insulin resistance in T2DM, the pathological transition of β-cells from dysfunction to failure is the core event of diabetes [109]. In that case, these β-cells can no longer serve as the source of stimuli for δ-cell secretion. Indeed, Ucn3 is greatly depleted from β-cells in diabetic mouse, macaque and human islets, which is in line with significantly reduced GISS [9] (Figure 5B). In α-cells, defective counter-regulation to hypoglycemia (Figure 5A) in T1DM and inappropriate postprandial glucagon release (Figure 5B) in T2DM are observed. The paracrine explanation for diminished responsiveness to hypoglycemia is later found to be excessive somatostatin secretion [10]. SSTR2 antagonists could improve α-cell counter-regulatory response [10,11] and ameliorate the incidence of hypoglycemia in T1DM animal models [110]. By contrast, in HFD mice, as well as in human T2DM islets [4], it has been reported recently that reduced somatostatin signaling, combined with intrinsic changes in α-cells, including insensitivity to somatostatin, collectively leads to hyperglucagonemia [111]. Thus, targeting the mechanisms responsible for the alternations in local communications would be promising for novel therapies that rebalance insulin and glucagon release.

## 8. Conclusions

Pancreatic δ-cells are emerging as vital “neighborhood patrols” that are well-positioned to maintain a well-functioning islet “community”. Although scarce in number, their elongated projections stretch their “jurisdiction” throughout the whole islet. On one level, δ-cells can sense multiple systemic signals, from “the superior—central nervous system” via neurotransmitters to “the ambient change” in circulating hormones and nutrients. After integrating the signals from multiple sources, they directly provide paracrine regulation to α- and β-cells to optimize glucagon and insulin secretion. More importantly, at the local level, δ-cells are highly sensitive to the chemical and electrical signals from intra-islet “residents”, α- and β-cells, and can release somatostatin accordingly to ensure little or no oversecretion of either insulin or glucagon. It is also fascinating that the plasticity of δ-cells may enable to them to establish local communications with non-δ-cells with different strength, in response to ever-changing environment. Overall, these highly interactive and plastic “communications” provide the basis for different pancreatic islet cells to integrate and form a functional unit for maintaining systemic glucose homeostasis.

In the setting of diabetes, the reduced detecting and regulatory power of δ-cells could result in “a chaotic islet community”: insufficient somatostatin secretion/signaling leads to oversecretion of insulin or glucagon and glucose volatility; oversensitive δ-cells or oversecretion of somatostatin, on the other hand, effectively paralyze hormone secretion from β-cells and α-cells, leading to hyperglycemia or hypoglycemia. Therefore, restoring appropriate δ-cell function or somatostatin secretion/signaling in diabetes could provide novel avenues for developing treatments to recover impaired islet function and improve glucose control in the disease.

## Data Availability

All the available data are presented in the article.
